# *Withania somnifera* (Ashwagandha) Improves Spatial Memory, Anxiety and Depressive-like Behavior in the 5xFAD Mouse Model of Alzheimer’s Disease

**DOI:** 10.3390/antiox13101164

**Published:** 2024-09-25

**Authors:** Noah Gladen-Kolarsky, Olivia Monestime, Melissa Bollen, Jaewoo Choi, Liping Yang, Armando Alcazar Magaña, Claudia S. Maier, Amala Soumyanath, Nora E. Gray

**Affiliations:** 1 Department of Neurology, Oregon Health and Science University, Portland, OR 97239, USA; 2 BENFRA Botanical Dietary Supplements Research Center, Portland, OR 97239, USAarmando.alcazarmagana@ubc.ca (A.A.M.);; 3 Linus Pauling Institute, Oregon State University, Corvallis, OR 97331, USA; 4 Department of Chemistry, Oregon State University, Corvallis, OR 97331, USA; 5 Life Sciences Institute, University of British Columbia, Vancouver, BC V6T 1Z4, Canada

**Keywords:** Alzheimer’s disease, memory, depression, anxiety, ashwagandha, neuroinflammation, oxidative stress, beta-amyloid, 5xFAD mice

## Abstract

*Withania somnifera* (WS), also known as ashwagandha, is a popular botanical supplement used to treat various conditions including memory loss, anxiety and depression. Previous studies from our group showed an aqueous extract of WS root (WSAq) enhances cognition and alleviates markers for depression in *Drosophila*. Here, we sought to confirm these effects in the 5xFAD mouse model of β-amyloid (Aβ) accumulation. Six- to seven-month-old male and female 5xFAD mice were treated with WSAq in their drinking water at 0 mg/mL, 0.5 mg/mL or 2.5 mg/mL for four weeks. In the fourth week of treatment, spatial memory, anxiety and depressive-like symptoms were evaluated. At the conclusion of behavioral testing, brain tissue was harvested, immunohistochemistry was performed, and the cortical expression of antioxidant response genes was evaluated. Both concentrations of WSAq improved spatial memory and reduced depressive and anxiety-related behavior. These improvements were accompanied by a reduction in Aβ plaque burden in the hippocampus and cortex and an attenuation of activation of microglia and astrocytes. Antioxidant response genes were upregulated in the cortex of WSAq-treated mice. Oral WSAq treatment could be beneficial as a therapeutic option in AD for improving disease pathology and behavioral symptoms. Future studies focused on dose optimization of WSAq administration and further assessment of the mechanisms by which WSAq elicits its beneficial effects will help inform the clinical potential of this promising botanical therapy.

## 1. Introduction

Alzheimer’s disease (AD) is the most common cause of dementia, accounting for 60–80% of all cases, and is the seventh leading cause of death worldwide [1]. An estimated 6.7 million Americans aged 65 and older are living with AD today, which could grow to as much as 13.8 million by 2060, barring the development of novel methods to prevent, cure or slow the disease [1]. In addition to the hallmark cognitive impairment associated with AD, studies have shown that the frequency of neuropsychiatric symptoms is also much higher in AD than in the general population, of which the most frequently observed symptoms are increased generalized apathy, anxiety and depression [2].

Pathologically, AD is characterized by the accumulation of β amyloid (Aβ) plaques and neurofibrillary tangles made up of hyperphosphorylated tau, which together contribute to synaptic dysfunction and eventual neuronal death [3]. Increased oxidative stress and chronic neuroinflammation are two additional consequences of plaque and tangle accumulation that are widespread throughout the AD brain [3,4].

Chronic neuroinflammation is central to AD progression and is believed to contribute to cognitive impairment [5]. Astrocytes and microglia work in tandem to mediate the inflammatory response to neuronal injury [6]. In early AD, these cells play a neuroprotective role, promoting cell repair, but as the disease progresses, they become chronically activated and produce pro-inflammatory cytokines, resulting in further neuroinflammation and neurotoxicity induced by inflammatory mediators [7,8,9]. Neuroinflammation is associated with reduced synaptic density and impaired cognitive function [10,11] and may also be a link between AD and depression [12,13] as studies have shown that treatments for neuroinflammation can improve endpoints related to depression [12,13].

Increased oxidative stress, caused by excess reactive oxygen species (ROS), is another early event in AD pathogenesis [14]. Rodent studies have demonstrated a direct relationship between oxidative stress and synaptic dysfunction in AD [15]. Antioxidant compounds can improve synaptic deficits in both in vitro and in vivo models of AD [16,17]. These alterations in synaptic function are thought to be the physiological underpinning of the improved cognitive function observed following antioxidant treatment in mouse models [18,19,20]. Although the mechanism is less well defined, increased oxidative stress is also linked with increased depression in patient populations [21].

Significant crosstalk also exists between the pathways mediating neuroinflammation and oxidative damage. Elevated ROS can trigger astrocytes and microglia to release pro-inflammatory cytokines in a coordinated response. These pro-inflammatory cytokines lead to further ROS production, as synaptic function and neurotransmission continue to deteriorate [9,22,23]. Because of this interconnectedness, there is growing interest in identifying therapeutic interventions that can target both inflammatory and antioxidant pathways.

Ashwagandha, or *Withania somnifera* (L.) Dunal (WS), is a traditional Ayurvedic herb widely used for treating an array of conditions, including memory loss, stress, anxiety, depression, and insomnia [24]. Extracts of WS were shown to improve cognitive function in various rodent models of neurodegeneration [25,26,27]. Antidepressive and anxiolytic properties were also reported in both rodent and clinical studies [24,28,29,30]. Our group has shown that an aqueous extract of WS (WSAq) can improve similar endpoints in *Drosophila* models as well [31,32]. We found that WSAq attenuated stress-induced depressive-like symptoms and improved performance in a phototaxis test in both healthy aged flies, as well as a fly model of increased oxidative stress [31,32].

Here, we evaluate the effect of WSAq in the 5xFAD mouse model of Aβ accumulation. The 5xFAD mouse model of AD overexpresses mutant human amyloid precursor protein (APP) containing the Swedish (K670N, M67IL), Florida (I716V), and London (V717I) Familial Alzheimer’s Disease (FAD) mutations, as well as the human presenilin 1 (PS1) gene with two FAD mutations, M146L and L286V. These mice model major pathological hallmarks resulting from Aβ accumulation as young as 2 months of age and develop robust plaque pathology along with severe cognitive impairment by 4–6 months of age [33,34]. In addition to assessing the behavioral effects on cognition, anxiety and depression elicited by WSAq, we also investigated the extract’s impact on Aβ plaque pathology and markers of oxidative stress and neuroinflammation.

## 2. Materials and Methods

### 2.1. WSAq Preparation and Chemical Analysis

WS root (Batch number 201000162) harvested in 2019 at Oregon’s Wild Harvest (Redmond, OR, USA) was obtained in powdered form and authenticated by genetic testing as previously described [35]. Voucher samples of the root powder are deposited at the Oregon State University Herbarium (voucher number OSC-V-265405) and in our laboratory (code number BEN-WS-8). Dried aqueous extract (WSAq) of the root was prepared as previously described [32]. Briefly, powdered root (160 g) was refluxed with boiling water (2 L) for 90 min. The extract was filtered while still warm and the filtrate cooled, frozen and lyophilized to yield a dry powder. Several batches of WSAq were made using a standardized protocol from the same starting root material (BEN-WS-8), and voucher samples are stored in our laboratory (code numbers BEN-WSAq-16, BEN-WSAq-18 and BEN-WSAq-19). BEN-WSAq-18 and BEN-WSAq-19 were used in feeding experiments. Targeted analysis of withanolides using liquid chromatography coupled to multiple reaction monitoring mass spectrometry (LC-MRM-MS) and chemical fingerprinting by untargeted analysis using liquid-chromatography coupled to high-resolution tandem mass spectrometry (LC-HRMS/MS) were performed on BEN-WSAq-16 using our published methods [35]. Marker withanolides determined by LC-MRM-MS (µg/g extract; reported are average ± S.D values derived from three technical replicates at 0.025 mg/mL plus three technical replicates at 0.25 mg/mL; n = 6 technical replicates): withanolide A (1157.70 ± 119. 88), withanone (832.52 ± 29.02), withaferin A (166.99 ± 14.14), withanoside IV (99.03 ± 6.02), 12-deoxywithastramonolide (50.91 ± 5.84), withanoside V (29.94 ± 2.28) and withanolide B (1.49 ± 0.12).

### 2.2. Animals

Experiments were carried out in line with the NIH Guidelines for the care and use of laboratory animals and were given approval by the Institutional Animal Care and Use Committee of the Veteran’s Administration Portland Health Care System (VAPORHCS; IACUC #4688-21). The 5xFAD and B6SJLF mice were purchased from Jackson Laboratory, Bar Harbor, ME, USA, and kept in a climate-controlled facility with a 12 h light/dark cycle. The 5xFAD transgenic male mice were paired with B6SJLF1 females to maintain the colony. Wild-type (WT) littermates were used as controls for each experiment.

**Figure 1:**
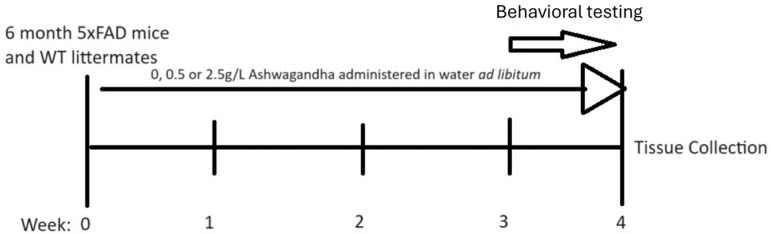
Treatment timeline for experiments.

At 6 months, male and female 5xFAD mice began treatment with either 0 mg/mL, 0.5 mg/mL or 2.5 mg/mL lyophilized WSAq extract administered via drinking water ad libitum for 4 weeks. Mice were not individually housed and, therefore, individual consumption could not be measured based on average consumption per cage adjusted for the number of mice in each cage and the average weight of all mice the doses correspond to approximately 75 mg/kg/day and 375 mg/kg/day. Age-matched WT littermates were treated with water containing 0 mg/mL for the same duration. Water was changed twice weekly to maintain water and additive quality. In the final week of treatment, mice underwent behavioral testing which included open field, then object location memory and lastly the forced swim test (Figure 1). Behavioral testing occurred only at the end of treatment (and not prior to and following treatment) in order to avoid the confounding effects of learning on performance in the task.

At the conclusion of behavioral testing, the mice were euthanized according to VAPORHCS guidelines and tissue was harvested. Experimental groups ranged from 13 to 15 animals per condition with slight variations between groups owing to litters that were not evenly divided between 5xFAD and WT (Table 1). All treatment groups included both male and female mice (Supplementary Table S1).

### 2.3. Object Location Memory Test (OLM)

The OLM test for spatial working memory was carried out in a square apparatus (39 cm × 39 cm × 39 cm). In the preliminary portion of the test, mice were habituated to the apparatus for 5 min per day for two days without any objects present in the field. On day 3, mice were introduced to the arena with two identical objects in fixed locations and were allowed to explore for three 10 min “training” intervals. Two hours and 24 h after the final training session, testing began, wherein mice were placed in the apparatus, but one of the two objects was moved from its original location to a new location in the field. The novel location was changed between the 2 h and the 24 h tests. Mice were then allowed to explore both objects for 5 min during each interval. The time spent exploring the novel location relative to the total time exploring both locations was scored manually by a blinded investigator using AnyMaze software version 6.13 and expressed as a percent. The objects used were of similar height and width. No preference was recorded in this study for any object. Increased time with the object in the novel location reflects improved spatial memory.

### 2.4. Open Field Test (OF)

The OF test is used to assess anxiety in mice. Animals are placed in a square arena (39 cm × 39 cm × 39 cm) and allowed to explore for 5 min. Time spent in different locations in the arena was quantified automatically using AnyMaze software. Increased time in the center of the arena indicates lower anxiety, while increased time spent in the periphery of the arena indicates higher anxiety [36].

### 2.5. Forced Swim Test (FST)

The FST evaluates depressive-like behavior. Mice are placed into a cylindrical container (45 cm in height, 20 cm in diameter) containing lukewarm water for 6 min and their time immobile is automatically scored using a camera and AnyMaze software. Greater time immobile is indicative of increased depressive-like behavior [37].

### 2.6. Immunohistochemistry

Right brain hemispheres were incubated in 4% paraformaldehyde for 24 h at room temperature and then transferred to phosphate-buffered saline (PBS), 15% and 30% sucrose solutions for 24 h each before being stored at −80 °C for sectioning. Coronal sections (40 µm) were obtained by slicing right hemisphere samples held at −20 °C in Optimal Cutting Temperature (O.C.T.) Compound (Sakura Finetek, Torrance, CA, USA) on a freezing microtome.

Sections were then stored in a sectioning solution (15% glycerol, 10% Tris-HCl buffered saline (TBS), diluted in diH_2_O) before further processing. During immunostaining, sections were placed in a quenching solution (30% methanol, 10% hydrogen peroxide, 10% TBS) for endogenous catalase activity and then blocked (2% bovine serum albumin, 10% horse serum, 2% triton x, 10% 10× TBS, diluted in diH_2_O). Sections were then incubated with one of the three following primary antibodies diluted 1:1000 in PBS: anti-AꞴ polyclonal antibody (Thermo Scientific, Waltham, MA, USA); GFAP (Glial fibrillary acidic protein; Invitrogen, Waltham, MA, USA); IBA1 (Ionized calcium-binding adaptor molecule 1; Proteintech, Rosemont, IL, USA); NRF2 (Abcam, Waltham, MA, USA) and visualized using biotinylated secondary antibodies.

ImageJ software (Rasband, W.S., ImageJ, version 1.54k) was used to perform the quantification of antibody staining. Images were converted to greyscale, and a tracing tool was used to outline the area of the cortex or hippocampus and the area outlined was noted. Contrast thresholding was adjusted according to background staining to highlight only intense staining. Staining was quantified for three coronal sections at different sectioning depths from each right hemisphere sample. Finally, the extent of staining was expressed as a percentage of area stained against the total area of the region in question, and mean values for each sample were calculated from the three sections analyzed.

### 2.7. Gene Expression Analysis

Left brain hemispheres were sub-dissected by brain region and frozen at −80 °C. RNA was extracted from one half of the cortex from the left hemisphere of each mouse brain using TRI reagent solution per the manufacturer’s protocol (Invitrogen). Reverse transcription was performed on the RNA product using a SuperScript™ III RT cDNA synthesis kit (Invitrogen) also per the manufacturer’s protocol. Quantitative PCR (qPCR) was performed using a QuantStudio™ 12K Flex Real-time PCR System (Applied Biosystems, Rosemont, IL, USA) using the following Taqman primers from Thermo: NRF2 (nuclear factor erythroid-derived 2-like 2, also called NFE2L2); HMOX1 (heme oxygenase 1); NQO1 (NAD(P)H quinone dehydrogenase 1); GCLC (Glutamate-Cysteine Ligase Catalytic Subunit); SYP (synaptophysin); PSD95 (post synaptic density protein 95, also called DLG4); GAPDH (glyceraldehyde-3-phosphate dehydrogenase). Relative expression was quantified using the delta delta CT method normalized to GAPDH expression.

### 2.8. Statistical Analysis

All bar graphs show error bars that reflect standard error of the mean. Statistical significance for graphed data was calculated using ANOVAs with Sidak pairwise post hoc testing. No interactions were found between sex and any outcomes measured and so results are presented with male and female mice together. All analyses were performed using GraphPad Prism version 10 software (GraphPad Software, Inc., La Jolla, CA, USA).

## 3. Results

### 3.1. WSAq Improves Spatial Memory in 5xFAD Mice

**Figure 2:**
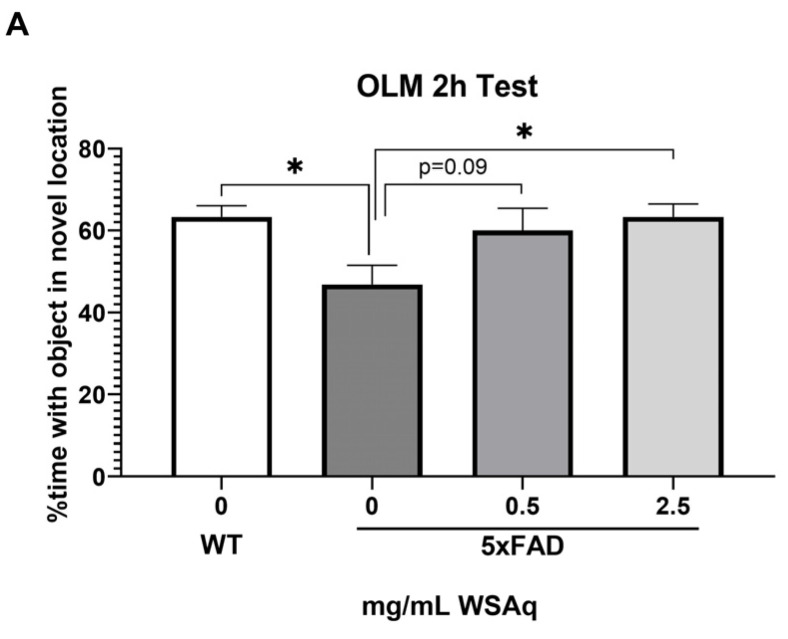

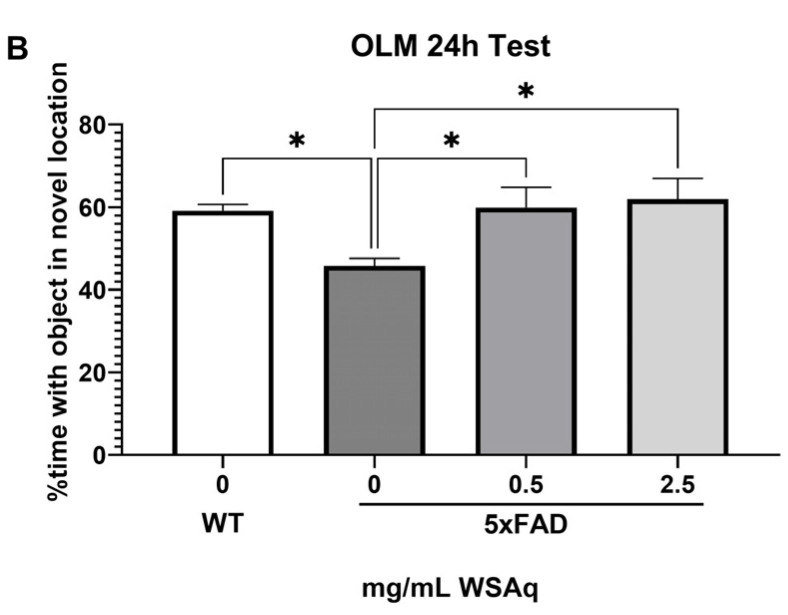
WSAq improves OLM performance in 5xFAD mice. WSAq treatment attenuated deficits in OLM performance at both 2 hours (**A**) and 24 hours (**B**). n = 9–12 per treatment group * *p* < 0.05.

In the OLM test, spatial memory retention was tested at 2 h (Figure 2A) and 24 h (Figure 2B) after the final training session. The 5xFAD mice receiving no WSAq spent significantly less time exploring the object in the novel location relative to their WT counterparts at both testing time points (Figure 2A,B). At the 2 h test, performance in the 5xFAD mice was significantly improved by 2.5 mg/mL WSAq treatment. There was a similar trend in the 0.5 mg/mL treated group, but it did not reach significance (Figure 2A). In the 24 h test, treatment with both concentrations of WSAq attenuated the deficit seen in the vehicle-treated 5xFAD mice (Figure 2B). At both time points, neither WSAq-treated group was significantly different from the WT mice.

### 3.2. Anxiety-Related and Depressive-like Behavior Is Reduced by WSAq in 5xFAD Mice

**Figure 3:**
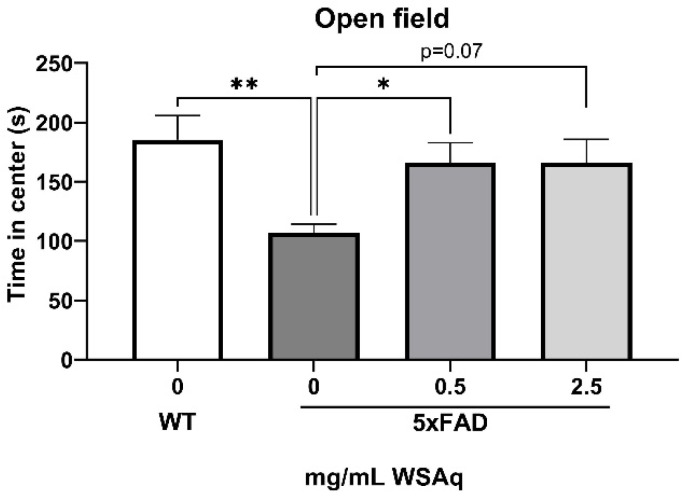
WSAq improves performance on the OF in 5xFAD mice. WSAq improved performance on the OF at the 0.5 g/L dose and approached significant improvement at the 2.5 g/L dose. * *p* < 0.05, ** *p* < 0.01. n = 8–12 per treatment group.

The 5xFAD mice showed increased anxiety compared to the WT mice, as measured by the reduced time in the center of the OF (Figure 3). WSAq treatment attenuated this decrease in time in the center to levels comparable to the WT mice. A significant increase in time in the center was observed in the 0.5 mg/mL group compared to the vehicle-treated 5xFAD mice and a similar but non-significant trend was seen with the 2.5 mg/mL-treated mice (Figure 3).

**Figure 4:**
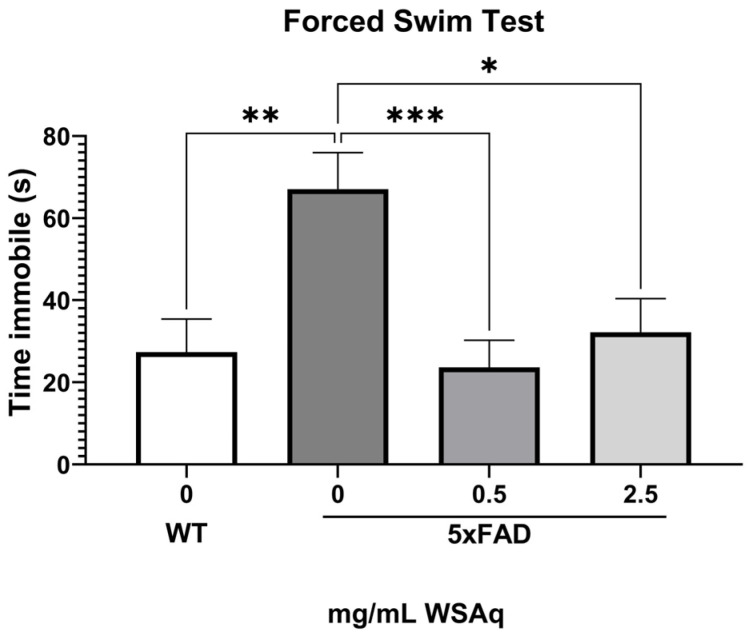
WSAq improves performance on the FST in 5xFAD mice. WSAq improved performance on the FST at both the 0.5 g/L and 2.5 g/L doses. WSAq treated groups were not significantly different from the WT group. * *p* < 0.05, ** *p* < 0.01, *** *p* < 0.001. n = 9–12 per treatment group.

During the FST, the 5xFAD control group spent significantly more time immobile than the WT group (Figure 4), indicating greater depressive-like behavior. Both concentrations of WSAq reduced time immobile to a similar extent in the 5xFAD mice (Figure 4).

### 3.3. Aβ Plaque Burden Is Reduced in WSAq-Treated 5xFAD Mice

**Figure 5:**
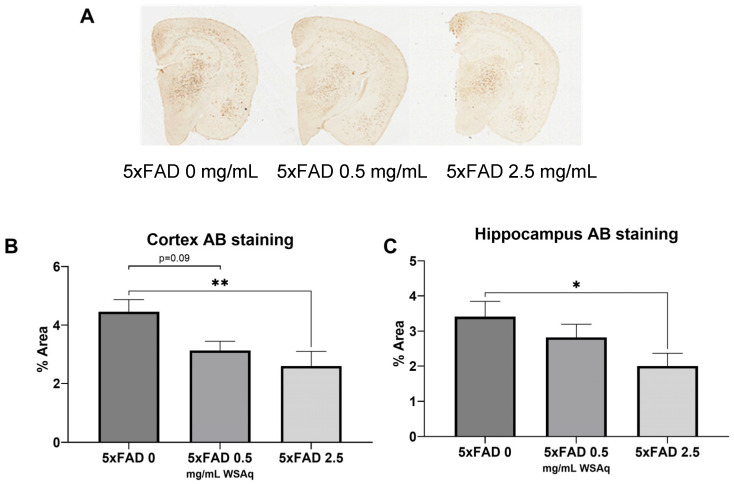
Aβ accumulation is attenuated following WSAq treatment. (**A**) Representative images from each 5xFAD treatment condition. In both the (**B**) cortex and (**C**) hippocampus a significant reduction of pan-Aβ staining at the 2.5 g/L dose was observed, * *p* < 0.05, ** *p* < 0.01. n = 13–15 per treatment group.

WSAq reduced the Aβ plaque burden in the 5xFAD mice (Figure 5A). In both the cortex (Figure 5B) and hippocampus (Figure 5C), 2.5 mg/mL WSAq reduced the plaque burden significantly compared to the 5xFAD mice that received 0 mg/mL WSAq. A similar, non-significant trend was also observed in the cortex of the WSAq 0.5 mg/mL-treated mice.

### 3.4. WSAq Reduces Astrocytic and Microglial Activation in 5xFAD Mice

**Figure 6:**
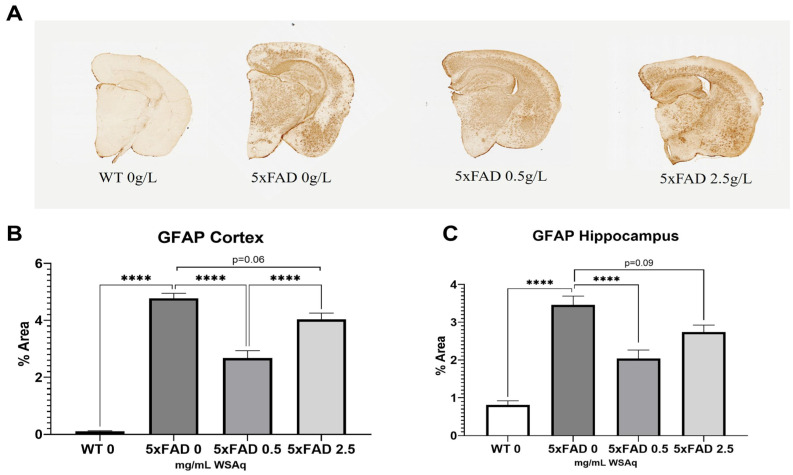
Astrocytic activation is reduced following WSAq treatment. Representative images of GFAP staining given by (**A**). A significant reduction in GFAP staining was observed at the 0.5 g/L dose but was not observed in the 2.5 g/L group in both the cortex (**B**) and hippocampus (**C**). **** *p* < 0.0001. n = 7–15 per treatment group.

Astrocytic activation was quantified via GFAP expression. The 5xFAD vehicle-treated mice had significantly higher GFAP expression than their WT littermates (Figure 6A). The 5xFAD mice treated with a 0.5 mg/mL dose of WSAq had a significant reduction in activated astrocytes in both the cortex (Figure 6B) and hippocampus (Figure 6C) as compared to the vehicle-treated 5xFAD mice. Interestingly, the same reduction was not seen in the 5xFAD mice treated with 2.5 mg/mL WSAq (Figure 6B,C).

**Figure 7:**
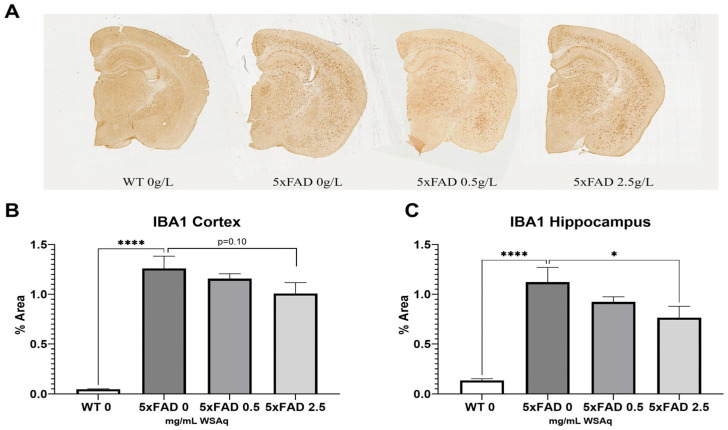
WSAq reduces microglial activation in 5xFAD mice. Representative images of Iba1 staining given by (**A**). Treatment with 2.5 g/L WSAq reduced activated microglia in the hippocampus (**C**) and approached significant reduction of IBA1 in the cortex (**B**). Activation following WSAq 0.5 g/L was not significantly different from the 5xFAD controls. * *p* < 0.05, **** *p* < 0.0001. n = 11–15 per treatment group.

The 5xFAD control mice also had significantly higher levels of microglial activation, as seen in IBA1 expression, than their WT counterparts (Figure 6A). The 2.5 mg/mL dose of WS reduced IBA1 staining significantly in the hippocampus of the 5xFAD mice (Figure 6C), and a similar trend was observed in the cortex (Figure 7B).

### 3.5. Antioxidant Response Is Upregulated in 5xFAD Mice Following WSAq Treatment

**Figure 8:**
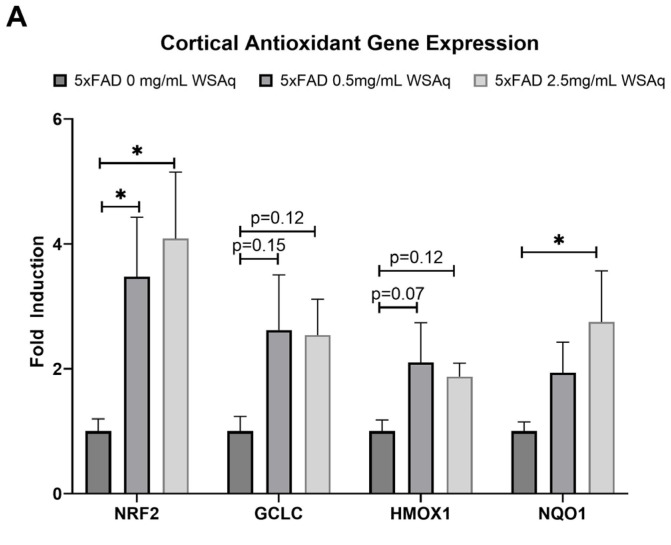

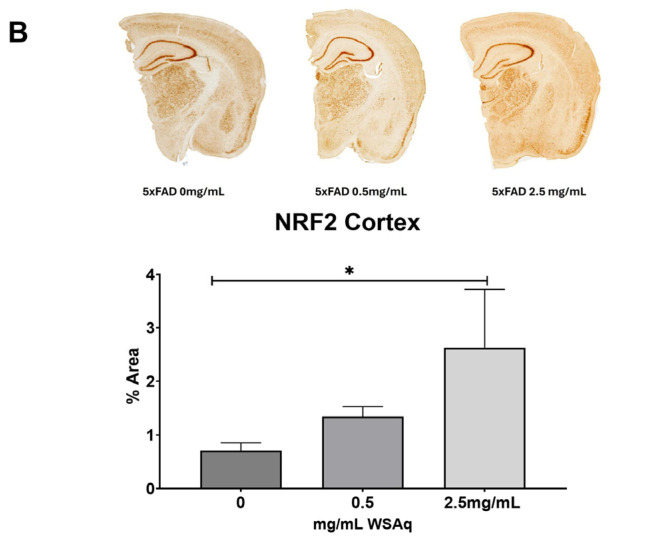
WSAq upregulates antioxidant response in the cortex of 5xFAD mice. (**A**) Gene expression of antioxidant response genes is increased in the cortex of WSAq treated 5xFAD mice. (**B**) NRF2 protein expression is likewise increased in the cortex of 5xFAD mice treated with 2.5 g/L WSAq. * *p* < 0.05. n = 9–12 per treatment condition.

An increase in the cortical expression of the antioxidant regulatory factor NRF2 was seen in the 5xFAD mice treated with both 0.5 mg/mL and 2.5 mg/mL WSAq (Figure 8A). There was also a significant increase in the cortical expression of the NRF2-regulated antioxidant enzyme NQO1 in the 5xFAD mice treated with 2.5 mg/mL WSAq. A similar trend towards increased expression of the NRF2-regulated antioxidant enzymes GCLC and HMOX1 was also seen in low and high WSAq-treated 5xFAD mice (Figure 8A). The protein expression of NRF2 was also increased in the cortex of the 5xFAD mice treated with 2.5 mg/mL WSAq (Figure 8B).

## 4. Discussion

This study investigated the effects of an aqueous extract of ashwagandha root (WSAq) in the 5xFAD mouse model of Aβ accumulation. Four weeks of oral treatment with WSAq resulted in improved spatial memory as well as reduced anxiety-related and depressive-like behavior. These changes were accompanied by a reduction in Aβ plaque burden and markers of neuroinflammation and increased antioxidant response.

The behavioral results from this study are in line with previous reports of the effects of WS extracts. Anxiolytic and anti-depressive effects of WS were demonstrated in both rodent and human models. In mice, WS root and leaf extracts increased mobility in the FST in Swiss albino mice [38] and oral treatment with a modified WS root extract ameliorated depressive and anxious behavior in a model of foot-shock-induced stress [30]. In humans, 8 weeks of daily administration of the product Sensoril**^®^**, containing both WS leaf and root extract, reduced stress, anxiety and depression in adults aged between 18 and 60 years old [39]. Similarly, 60 days of treatment with WS root extract standardized for 2.5% of the withanolide compounds found in WS improved stress and anxiety metrics in healthy adults exhibiting mild to moderate symptoms of those endpoints [40]. The same duration of treatment with the product Shoden**^®^**, containing a WS root and leaf extract standardized to 35% withanosides, also improved scores for anxiety and stress in healthy adults [28]. Similar results were seen in stressed adults as well. Improvements in anxiety and stress scores were also seen in stressed adults given Shoden**^®^** [28] and in adults reporting a history of chronic stress, WS extracts were reported to significantly reduce stress assessment and anxiety scores [41,42].

Preliminary studies using WS in human trials have also provided evidence for its cognition-enhancing capabilities. A systematic review of five studies exploring the effect of WS on cognition found evidence for improved executive function, attention, reaction time, and cognitive task performance, but low-quality studies and heterogeneity of the study populations limit the impact of these findings [43]. Stronger evidence of cognitive enhancement elicited by WS was reported in pre-clinical models. Oral treatment with various WS preparations was shown to improve cognitive deficits in rodent models of scopolamine-, hypoxia- and high-fat diet-induced cognitive impairments [27,44].

Despite the evidence for cognitive-enhancing, anxiolytic and anti-depressive effects of WS, the exact molecular mechanisms through which these behavioral changes are elicited remain poorly defined. In the present study, the behavioral improvements may be related to the reduction in Aβ plaque burden in WSAq-treated 5xFAD mice. This finding is consistent with existing literature on the effects of WS on Aβ [45,46,47]. A similar plaque reduction was reported in the APPswe, PSEN1 transgenic model of Aβ accumulation following a 30-day treatment period with a powdered root extract suspended in ethanol [48]. The study proposed a unique mechanism by which WS affects Aβ clearance from the brain into the periphery by modulating liver production of low-density lipoprotein receptor-related protein (LRP) which traffics Aβ from the brain [48]. There have also been in vitro reports of constituent compounds from WS reducing the levels of Aβ by inhibiting Aβ secretion [49,50]. Future studies are needed to determine whether the Aβ lowering effect of WSAq is the result of altered production or clearance in 5xFAD mice. The effects of WS on Aβ are interesting in light of the newer FDA approved Aβ lowering therapies [51,52]. While these therapies are major breakthroughs there are still limitations including a narrow window to initiate treatment in a specific population of AD patients as well as some safety concerns [53]. WSAq may prove to be an alternative that does not suffer from the same limitations, although that remains to be seen as it continues to be developed for clinical testing.

In this study, there was a potent effect of WSAq on microglial and astrocytic activation in the 5xFAD mouse brain. These findings are in line with other studies that have demonstrated the anti-inflammatory effects of WS [47,54,55,56]. A sustained release formulation of WS, AshwaSR, at a dose of 100 mg and containing NLT 5% total withanolides administered once daily to Sprague Dawley rats inhibited the expression of pro-inflammatory cytokines IL-1β and TNF-α, and in an in vitro portion of the study, AshwaSR showed dose-dependent inhibition of IL-1B and TNF-α production from LPS-induced THP-1 human monocytes [57]. Further evidence is also seen in rodent models. In young adult female Wistar rats with diet-induced obesity, WS dry leaf powder reduced the expression of GFAP and IBA1 in the hippocampus along with other inflammatory markers [58]. Similarly, GFAP expression was reduced in adult male Albino Wistar rats treated with a fine leaf powder extract of WS [59]. Although the effects of WS on neuroinflammation in humans have not been thoroughly investigated, there is evidence for the anti-inflammatory effects of WS in numerous other conditions including lupus erythematosus, inflammatory bowel disease, rheumatoid arthritis and coronavirus [60]. It is possible that the anti-inflammatory effects of WS may be mediators of the behavioral effects observed in this study, as neuroinflammation has been linked to affective and cognitive dysfunction [61]; however, more work is needed to confirm this mechanistic link which could include a quantitative assessment of other inflammatory markers and more in-depth microglial and astrocytic profiling.

Interestingly, the effects of WS on inflammation are not unequivocally positive. The pro-inflammatory effects of WS preparations were also reported in the literature. Increased GFAP expression was seen in scopolamine-exposed Swiss albino mice treated with an alcoholic extract of WS leaves (i-Extract) at 100, 200 and 300 mg/kg [62]. One possible reason for these divergent findings could be that the concentrations used were much higher than the concentration of WSAq used in the present study. It is possible that the maximal anti-inflammatory effects of WS are evoked at lower concentrations and once those concentrations are exceeded the effects diminish or are even reversed. It is also possible that the pro-inflammatory compounds of WSAq only attain active concentrations at higher doses. In fact, the GFAP data from the current study support this idea, as the reduction in expression was only observed in 5xFAD mice treated with 0.5 mg/mL and not 2.5 mg/mL of WSAq. The fact that lower concentrations of WS may confer greater benefits than higher concentrations of WS is noteworthy especially in light of recent reports of liver toxicity following WS administration [63]. Taken together, these findings underscore the importance of precise dosing of WS for optimal effects on inflammation.

WSAq also induced the expression of antioxidant response genes and the expression of the antioxidant regulatory protein NRF2 in the cortex of treated 5xFAD mice in the present study which could also have contributed to the behavioral improvements observed. The antioxidant effects of WS were also reported in other model systems as well. Cultured BV-2 microglial cells treated with the WS constituent compounds withaferin A and withanolide A showed reduced LPS-induced NO production and activation of the NRF2 antioxidant response pathway [64]. Similar effects were observed in vivo. WS powdered root extract attenuated MPTP-induced deficits in superoxide dismutase (SOD) and catalase (CAT) in albino mice [65] and normalized malondialdehyde (MDA), SOD and glutathione (GSH) activity in 5xFAD mice [66]. WS was also found to exert an antioxidative effect in human trials. In a study of healthy adults treated with dried aqueous root extract for 6 months, a significant increase in SOD was observed [67]. Other markers of antioxidant response and oxidative stress have similarly been reported to be altered by WS in humans, including decreased levels of MDA and nitric oxide and increased expression of GSH, SOD, and CAT enzymes [68]. The antioxidant effects of WSAq could be confirmed in future studies by evaluating the protein expression of the antioxidant enzymes and markers of oxidative damage.

It is important to note that many WS preparations discussed in the literature are complex mixtures of phytochemical compounds as is WSAq. While some preparations are standardized to specific levels of constituent compounds, it remains to be seen which chemical constituents mediate the beneficial effects of the extracts in the context of neurodegeneration. Withaferin A was shown to improve cognitive function in a mouse model of frontotemporal lobar degeneration [69] and sominone, a metabolite of withanoside IV, enhanced location memory in healthy young mice [70]. Continued research into the anti-depressive and anxiolytic properties of constituent compounds of WS and the molecular mechanisms underpinning the observed behavioral effects will be valuable in understanding how WS is able to confer its beneficial effects.

It is notable there was no difference in response to WSAq observed between male and female 5xFAD mice. This study was designed to evaluate the effects of WSAq in animals with robust Aβ pathology. It would be interesting in future studies to explore the effects of an earlier intervention to determine if WSAq treatment could prevent or delay the onset of pathology. It will be important in such a study to again take into account the effects of sex in light of the differential risk based on sex of developing AD that exists in the human population.

## 5. Conclusions

The findings from this study support the therapeutic potential for WSAq to improve cognition and reduce anxiety and depressive symptoms in the context of AD. Future studies are needed to more fully understand the cellular mechanism by which WSAq exerts these beneficial effects and to identify optimal dosing for behavioral improvements. Because neuroinflammation and oxidative stress accompany cognitive impairment, anxiety and depression in other conditions beyond AD, the results presented here suggest the potential for a broader application of WSAq to other neurodegenerative conditions as well as to support healthy aging.

## Figures and Tables

**Table 1:** Number of animals per experimental group.

	0 mg/mL	0.5 mg/mL	2.5 mg/mL
WT	14	--	--
5xFAD	15	13	13

## Data Availability

The datasets used and/or analyzed in the current study are available from the corresponding author upon reasonable request.

## Supplementary Materials

The following supporting information can be downloaded at: https://www.mdpi.com/article/10.3390/antiox13101164/s1. Table S1: Number of male and female mice in each treatment condition.
