# Supplementary material for: Withania somnifera (Ashwagandha) Improves Spatial Memory, Anxiety and Depressive-like Behavior in the 5xFAD Mouse Model of Alzheimer’s Disease
Source: Antioxidants (Basel). 2024 Sep 25;13(10):1164. doi: 10.3390/antiox13101164 (PMC11504317; doi:10.3390/antiox13101164)
Supplement: Supplementary file 1 [file antioxidants-13-01164-s001.zip › antioxidants-3183625-supplementary.pdf]

antioxidants

Table S1. Number of male and female mice in each treatment condition.

	WT	5xFAD		
	Control	Control	0.5mg/mL	2.5mg/mL
Females	7	6	7	5
Males	7	9	6	8
